# CRISPR-Cas9: A Powerful Tool to Efficiently Engineer *Saccharomyces cerevisiae*

**DOI:** 10.3390/life11010013

**Published:** 2020-12-26

**Authors:** João Rainha, Joana L. Rodrigues, Lígia R. Rodrigues

**Affiliations:** Centre of Biological Engineering, University of Minho, 4710-057 Braga, Portugal; joao.rainha@ceb.uminho.pt (J.R.); joanarodrigues@ceb.uminho.pt (J.L.R.)

**Keywords:** CRISPR-Cas9, CRISPR-Cas9 applications, genome editing, *Saccharomyces cerevisiae*

## Abstract

*Saccharomyces cerevisiae* has been for a long time a common model for fundamental biological studies and a popular biotechnological engineering platform to produce chemicals, fuels, and pharmaceuticals due to its peculiar characteristics. Both lines of research require an effective editing of the native genetic elements or the inclusion of heterologous pathways into the yeast genome. Although *S. cerevisiae* is a well-known host with several molecular biology tools available, a more precise tool is still needed. The clustered, regularly interspaced, short palindromic repeats–associated Cas9 (CRISPR-Cas9) system is a current, widespread genome editing tool. The implementation of a reprogrammable, precise, and specific method, such as CRISPR-Cas9, to edit the *S. cerevisiae* genome has revolutionized laboratory practices. Herein, we describe and discuss some applications of the CRISPR-Cas9 system in *S. cerevisiae* from simple gene knockouts to more complex processes such as artificial heterologous pathway integration, transcriptional regulation, or tolerance engineering.

## 1. Introduction

*Saccharomyces cerevisiae* has long been a common model for fundamental biological studies because of its safety and ease of handling. This yeast has been extensively used to elucidate eukaryotic processes from basic metabolism to protein/gene interaction or even evolution [1]. Moreover, it is also a popular biotechnological engineering platform used to produce chemicals, fuels, and pharmaceuticals [2]. Associated with these two lines of research is the need to effectively edit the native genetic elements or introduce heterologous pathways into the yeast genome. For instance, for metabolic engineering purposes, several genetic modifications are required besides the introduction of heterologous genes in order to stream the metabolic fluxes toward the production of the desired product. The development of an efficient cell factory is associated with several native gene deletions, overexpression, or replacements. All these modifications require a cycle of individual transformation, selection, and confirmation, thus making the process very time-consuming. Furthermore, each cycle is associated with the integration of a selective marker. However, the number of markers available is limited, which also limits the number of sequential modifications that can be performed.

*S. cerevisiae* is known to possess a very efficient homologous recombination (HR) machinery [3]. Researchers have been taking advantage of this feature for in vivo assembly of multiple linear fragments instead of using in vitro molecular biology techniques. Most of the in vivo assembly performed in yeast was focused on circular vector construction. However, for metabolic engineering purposes, genomic integration offers a more stable expression system and eliminates the need of ensuring a selective pressure for plasmid maintenance. Nevertheless, in vivo assembly in combination with genomic integration is associated with very low efficiencies [4].

Double-strand breaks (DSBs) in the genome occur due to several environmental factors and can be repaired by HR or nonhomologous end-joining (NHEJ). In *S. cerevisiae* it is known that the dominant repair is performed by HR. The introduction of a DSB has been shown to increase the efficiency of homologous integration of linear DNA fragments with homologous ends [5]. These features led researchers to develop methods that use DSBs for site-directed genome editing. Theoretically, these methods could be used for marker-free modifications and examples include zinc finger nucleases (ZFN) and transcription-activator-like effector nucleases (TALEN). Both methods involve the design of proteins that recognize a specific DNA sequence and cause a site-specific DSB through DNA–protein interaction. The DSB is then repaired by HR with a provided DNA fragment with homologous ends. However, a new ZFN or TALEN protein has to be engineered for each target modification, making these methods also very laborious [6,7].

Evolution allowed bacteria to develop several systems to degrade foreign DNA as a defense mechanism. The most well-known are restriction enzymes, which rapidly became the “workhorses of molecular biology” [8]. More recently, another prokaryotic immune system was identified [9]. This system consists of clustered, regularly interspaced, short palindromic repeats (CRISPR) and CRISPR-associated nucleases (CRISPR-Cas), widely distributed among bacteria and archaea [10]. CRISPR-Cas has the function of recognizing and degrading invading nucleic acids. The system is divided in three classes (I, II, and III) based on the *cas* gene sequences, operon organization, and the repeats within CRISPR arrays [11]. The increased knowledge of CRISPR action mechanism broke new ground regarding a possible applicability for this system to edit genetic elements of an organism.

**Figure 1:**
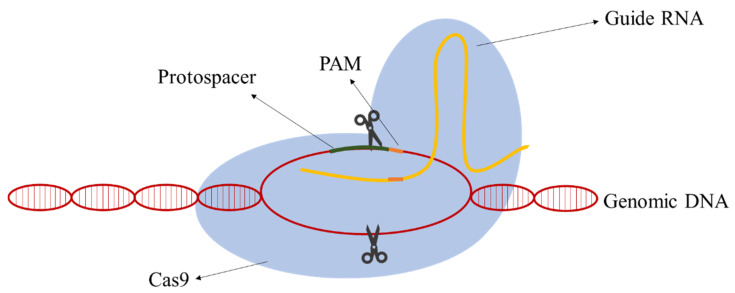
The mechanism of action of clustered, regularly interspaced, short palindromic repeats–associated Cas9 (CRISPR-Cas9). The guide RNA (gRNA) molecule directs Cas9 protein to the target DNA and Cas9 cleaves genomic DNA 3–4 bp upstream of the PAM (protospacer-adjacent motif) site.

Due to their simplicity relative to other classes, class II CRISPR-Cas systems have the most well-developed methods for genomic engineering nowadays. In class II, all the functions of the effector complex are performed by a single protein [12]. The following components are required: the RNA-guided nuclease Cas9, a CRISPR RNA (crRNA), an auxiliary trans-activating crRNA (tracrRNA), and an RNase III. The action mechanism is simple and aims at the creation of a DSB in the target DNA. A hybrid of the RNA molecules directs Cas9 to the target DNA site and the Cas9 cleaves a targeting DNA sequence containing a protospacer-adjacent motif (PAM) sequence. Jinek et al. [13] postulated that the requirement for RNase and tracRNA can be bypassed by fusion crRNA and tracrRNA to form a single-guide RNA (gRNA) simplifying the process and application. The gRNAs are composed of a homologous sequence 20 nt upstream of the PAM sequence (guide sequence) and the scaffolding loop structure to attach the Cas9 (Figure 1).

The type II CRISPR-Cas9 system was implemented in *S. cerevisiae* for genetic modification purposes by DiCarlo et al. [14]. The researchers demonstrated that co-transforming a gRNA targeting a negative selectable marker together with a 90-bp double-stranded HR donor with a frameshift mutation in the targeted reading frame and PAM replacement by a stop codon resulted in almost 100% of mutated cells. 

In this review, we discuss in detail the CRISPR-Cas9 system components with a focus on the *S. cerevisiae* chassis, as well as the recent CRISPR-Cas9-based applications. 

## 2. The CRISPR-Cas9 System Components

### 2.1. Cas9 Protein

The Cas9 protein from *Streptococcus pyogenes* (*Sp*Cas9) fused to a nuclear tag is the most used Cas9 protein in *S. cerevisiae*. The chosen Cas9 influences the guide RNA design because Cas9 from different origins recognize different PAM sequences. For instance, *Sp*Cas9 recognizes the PAM sequence (5’–3’) NGG. The DNA sequence of Cas9 used in yeast can be native, codon-optimized for yeast or even for humans [14]. Regarding the expression system, usually Cas9 is expressed under the control of constitutive promoters from self-replicating centromeric (e.g., [14]) or 2µ (e.g., [15]) plasmids. Other studies integrated the Cas9 in yeast genome (e.g., [16]). Cas9 toxicity has been reported in some experiments, however this can be easily bypassed using weaker or inducible promoters [14,17]. The Cas9 expression system can be chosen to fit the particularities of the research. For instance, a plasmid-based strategy is preferred for single modifications because the plasmid can be easily cured after the process. On the other hand, for multiple modifications, the genomic integration offers a more stable platform for Cas9 expression.

### 2.2. Guide RNA

The key step for the development of an efficient CRISPR-Cas9 system to target a desired genomic sequence is the design, expression, and delivery of the RNA components. As already mentioned, in *S. cerevisiae* the most popular strategy has been the expression of a single chimeric RNA molecule named gRNA. The gRNA combines the functions of crRNA and a tracRNA in a single RNA complex making the construction and application easier. To ensure the right function of gRNA to form a Cas9/gRNA complex, both ends must be accurately defined. For the expression of gRNA in *S. cerevisiae,* RNA polymerase III promoters are commonly used. The expression cassette containing the small nucleolar RNA (SNR52) promoter, an RNA polymerase III promoter, and the SUPpressor (SUP4) terminator was used by DiCarlo et al. [14] to functionally express the gRNA in a laboratory yeast strain with engineering efficiencies reaching 100%. The same system was also used in industrial strains with efficiencies ranging between 65% and 78% [18]. Nevertheless, other cassette setups were also used yielding good results. For instance, Ryan et al. [19] expressed the gRNA fused to a Hepatitis delta virus ribozyme controlled by a tRNA promoter and SNR52 terminator. The efficiency was almost 100% in a laboratory strain and 90% in an industrial strain. It was observed that the presence of the fused ribozyme increased the number of synthesized gRNAs. The authors reasoned that the ribozyme would protect the 5’ end of gRNA from endogenous 5’ exonucleases resulting in higher efficiencies. RNA polymerase II promoters were also used achieving 100% efficiency [20]. In contrast, other researchers separated the expression of the targeting crRNA and tracRNA instead of using the chimeric gRNA. Both RNA molecules were transcribed by different RNA polymerase III promoters. The efficiencies obtained ranged from 75% to 100% [15]. 

**Figure 2:**
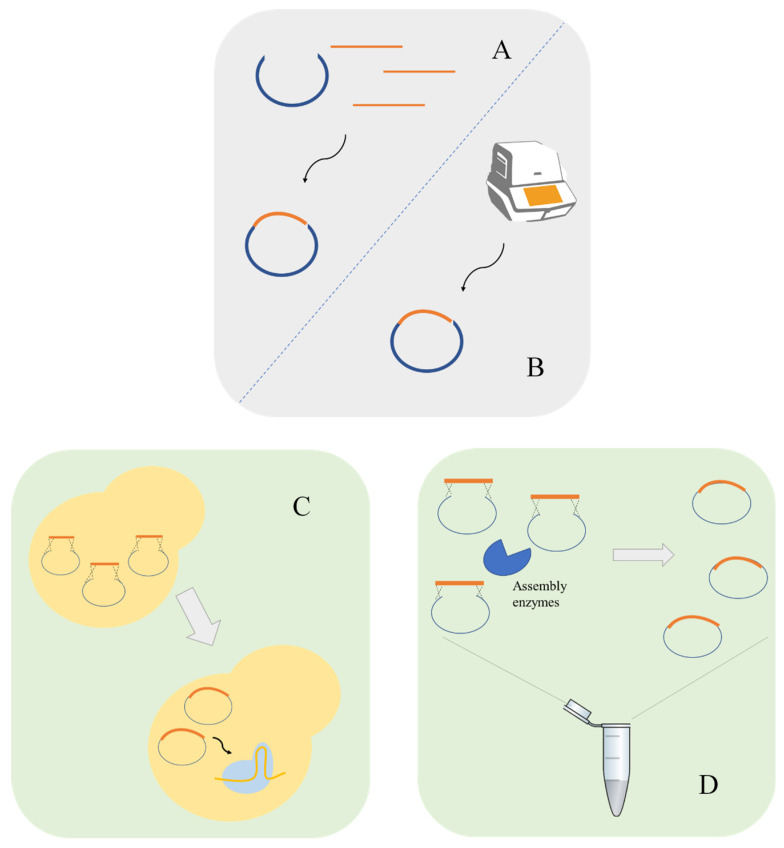
Molecular biology techniques for the construction of guide RNA (gRNA) carrying plasmids. gRNA plasmids can be constructed using traditional methods such as digestion–ligation using restriction enzymes and T4 ligase (**A**) or by PCR based methods such as circular polymerase extension cloning (CPEC) (**B**). Homology-recombination-based methods are other possible approaches. Homology recombination can be performed by in vivo assembly using *S. cerevisiae* machinery (**C**) or using in vitro methods such as Gibson assembly (**D**).

The gRNA molecule is composed by a scaffolding sequence, necessary for Cas9 binding, and by the guide sequence. The design of a gRNA to target a defined locus requires the ~20 bp guide sequence upstream of the selected PAM sequence to direct Cas9. The vector construction containing the desired gRNA is another challenge in the development of CRISPR systems. Most studies use PCR to insert the gRNA sequence using phosphorylated primers containing the target-specific 20 bp sequence to amplify the whole vector followed by recircularization via ligase [21]. Cloning methods such as circular polymerase extension cloning (CPEC), which uses polymerase to join overlapping DNA fragments can also be used to clone gRNA in a plasmid [22]. Fragment assembly is another possible approach, these can be accomplished by in vivo assembly [17] or by in vitro methods such as the Gibson assembly [23]. In addition, restriction enzymes located between the promoter and the gRNA scaffolding sequence can also be used [24]. To avoid PCR amplifications, the gRNA cassette can be synthesized and integrated in vectors using for instance Uracil-Specific Excision Reagent (USER) cloning [25] or Golden Gate Assembly [15] (Figure 2).

Since any ~20 bp sequence next to a PAM site can be used in the gRNA design, the biggest concern is to ensure that the Cas9 will not recognize an unwanted sequence in the genome leading to off-target effects. The off-target effect, when a DNA site other than the target is cleaved by Cas9, must be considered when performing genetic modifications using the CRISPR-Cas9 system. Other factors may influence the gRNA design and may vary according to the desired application. For instance, for gene knockout, the beginning of an open reading frame (ORF) is preferred to insert a STOP codon to precociously stop transcription avoiding any protein translation. Regarding the choice of guide sequence, there is a wide collection of online tools available to help researchers design a gRNA for a target genomic sequence. The tools aim to minimize the off-target effects by matching the desired sequence against the reference genome assigning a score based on the specificity of the guide sequence or by looking for potential PAM sites with minimized possible off-targets in a given DNA sequence. Some of the tools also provide other potential requirements to ensure an efficient DSB, such as the GC content and self-complementary or poly T presence in the gRNA molecule, calculating a score also based on these features. Some available webtools include, CRISPy (www.crispy.secondarymetabolites.org) [26], CRISPR-ERA (www.crispr-era.stanford.edu) [27], E-CRISPR (www.e-crisp.org) [28], Benchling (www.benchling.com), or ATUM gRNA design tool (www.atum.bio). 

However, since *S. cerevisiae* has a high HR capability and NHEJ rarely occurs, the chances of off-target effects is drastically reduced as confirmed in some works [14,21]. Nevertheless, the high efficiencies reported are only applied for laboratorial yeast strains, which are usually haploid or diploid homozygotic making possible the performance of markerless genetic modifications carried out by the CRISPR-Cas9 system. In heterozygous organisms, such as polyploid industrial *S. cerevisiae* strains or other eukaryotic organisms, a gRNA can be designed for an allele-specific target if a loci has different PAM sites [29]. However, the presence of an intact chromosome could facilitate the HR repair competing with the editing event in a phenomenon called loss of heterogenicity (LOH). De Vries et al. [30] observed that the editing efficiency can decrease by two orders of magnitude when targeting only one of two homologous chromosomes in a heterozygous *S. cerevisiae*. The authors suggested that gRNAs should target homozygous stretches in heterozygous genomes, and markers should be used when allele-specific gene editing is required. Methods for the identification of CRISPR-Cas9 off-target sites have been developed and are reviewed elsewhere [31]. However, these types of studies are usually employed in relatively more complex genomes such as mammalian cells. Notwithstanding, Waldrip et al. [32] used cross-linking chromatin immunoprecipitation (ChIP) sequencing to detect off-target sites in *S. cerevisiae* and found that, as expected, Cas9 is highly specific and virtually lacks off-target sites within the yeast genome. 

### 2.3. Donor DNA 

As mentioned, the dominant mode of DSB repair in yeast is HR. In order to efficiently apply a CRISPR-Cas9 system to a desired genomic target, it is necessary to supply the homologous donor template carrying the desired modification. The use of short single-strand [17] or double-strand [14] DNA donor oligos can act as the simplest repair template. The lengths on donor DNA vary depending on the type of desired modification, from a STOP codon insertion to a complete heterologous biosynthetic pathway. It is very important to ensure that, after recombination, the PAM site is removed from the target to prevent continuous cutting by Cas9. Regarding the donor delivery, it can be as part of an expression vector [15] or as simple linear DNA oligos. The CRISPR system was also combined with in vivo assembly of various DNA fragments eliminating the need of cloning processes. For instance, a metabolic pathway of six genes and 300 bp homology arms consisting in four DNA fragments was assembled in vivo and used as a DSB repair template by Tsai et al. [33]. Three DNA oligos carrying a genetic pathway and 1 kb homologous arms were used by Apel et al. [23] resulting in 40% efficiency in a determined locus. Although versatile and faster, the combination of donor in vivo assembly with CRISPR-Cas9 DSB repair is associated with low efficiencies. Pre-assemble of the DNA fragments always leads to higher efficiencies [34]. Plasmids containing combined gRNA and a DNA repair template can also be used [35]. Given that during gRNA or crRNA maturation, the 5’ end region is cleaved by 5’ exonuclease and other endogenous nucleases, the researchers hypothesized the use of this region to harbor the HR disruption donor DNA, as seen in some works [15]. This is advantageous when the combined DNA elements are small enough to be synthesized and inserted into the CRISPR plasmid. 

Regarding the length of homology arms, the minimal homology required is not directly related with the CRISPR-Cas9. Early studies in genetic integration defined 30 bp minimal homology at each end of a DNA fragment for recombination [36]. However, higher homology arms (200 to 1000 bp) result in high yield of recombination efficiencies mainly when large heterologous pathways need to be integrated.

## 3. CRISPR-Cas9 Applications in *Saccharomyces cerevisiae*

### 3.1. Knockin and Knockout 

Simple knockin or knockout of genes are routinely used in *S. cerevisiae* studies of fundamental metabolism or also to develop novel strains adapted to produce useful chemicals. These modifications are easily achieved by conventional gene recombination, due to the high HR capability of this yeast [37], however they are inherent to the use of selective markers. Although recombinase-based methods such as Cre/LoxP or Flp/FRT have been extensively used in marker recycling [38,39], they are time-consuming since they require another transformation cycle to remove the marker. In addition, these methods have the drawback of leaving a copy of a repeat sequence in the genome. These “scars” may lead to genome instability after multiple rounds of marker recovery [40]. The development of the CRISPR-Cas9 system allowed the researchers to perform high-efficiency knockin and knockout without the use of selection markers. Different studies have proved that single and multiple gene disruptions/integrations can be achieved by the combinatorial effects of high-efficiency HR and CRISPR system in a markerless way. The system has proven to be very efficient with values ranging up to 100%. 

**Figure 3:**
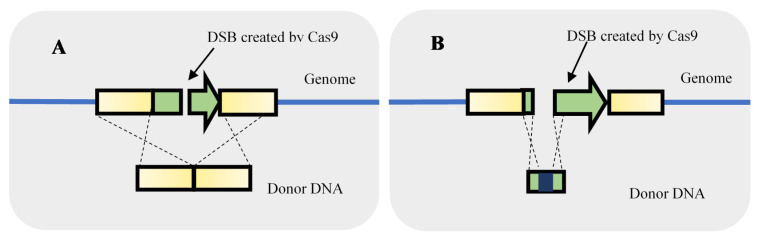
Strategies used to gene knockout using double-strand breaks (DSB) originated by clustered, regularly interspaced, short palindromic repeats–associated Cas9 (CRISPR-Cas9). The knockout can be performed by providing a homologous repair fragment for the deletion of an entire gene (**A**) or to cause a frameshift modification (**B**).

Regarding the knockout of genes, Generoso et al. [17], for instance, designed a CRISPR-Cas9 system to create a DSB in the threonine deaminase (ILV) gene. The single-stranded donor DNA used for recombination was composed of 40 nt upstream and 40 nt downstream of the ILV ORF, the knockout was 100% efficient. No marker integration was required to delete an entire ORF. For gene knockout, besides deleting an entire ORF, other strategies can be used such as frameshift modification or insertion of a STOP codon (Figure 3). To knockout the canavanine resistance (CAN1) gene, Bao et al. [15] caused a 8 bp deletion leading to a frameshift resulting in CAN1 knockout by putting a STOP codon in the frame in the beginning of the gene. The 8 bp included the PAM site and the last 3 bp of the gRNA to prevent the continuous recognition and cleavage by the CRISPR-Cas after recombination.

Multiple gene disruptions can be used for gene characterization in complex genetic pathways [41] and also to facilitate the development of an efficient chassis for heterologous production [42]. Sequential gene disruptions are traditionally achieved by the replacement of the target gene with a selectable marker through HR in a sequential manner. The markers are usually recycled by different systems, which is a very laborious process. CRISPR-Cas9 can bypass this limitation, however there are some problems associated with multiple gRNAs’ expression, mainly the need to use various RNA polymerase III regulatory elements to express them. Bao et al. [15] developed a CRISPR system to overcome this limitation. For that purpose, the expression of three gRNAs was carried by a single promoter and the sequences were separated by direct repeats. In a different experiment, in order to knockout three genes in a single step, Generoso et al. [17] expressed the gRNAs separately, also obtaining good results. Jakočinas et al. [21] developed a CRISPR system for quintuple gRNA expression in a single plasmid controlled by a single SNR52 promoter. Good efficiencies were reported, however the transformation efficiency decreased with the number of expressed gRNAs. Liu et al. [43] expressed three gRNAs in a single cassette and achieved triple disruption efficiencies of 95%. Therefore, for the expression of multiple gRNAs using a single RNA polymerase promoter, each gRNA should be flanked by cleavage sequences such as direct RNA repeats [15]; self-cleavable ribozyme sequences [19,20] or by RNA processing sequences such as tRNA. Zhang et al. [44] developed a multiplex CRISPR system expressing gRNA–tRNA arrays with a single RNA polymerase promoter. The researchers reported a simultaneous disruption of eight genes, which is currently the highest multiplex edition reported in yeast.

Direct point mutations (single-nucleotide changes) can also be performed using CRISPR-Cas9. Mans et al. [16] efficiently caused point mutations in specific genes using a CRISPR approach. This kind of approach can be useful for studies of the biological significance of mutations caused by artificial evolution, since not all mutations obtained in this kind of experiments contribute to a desired phenotype [45].

**Figure 4:**
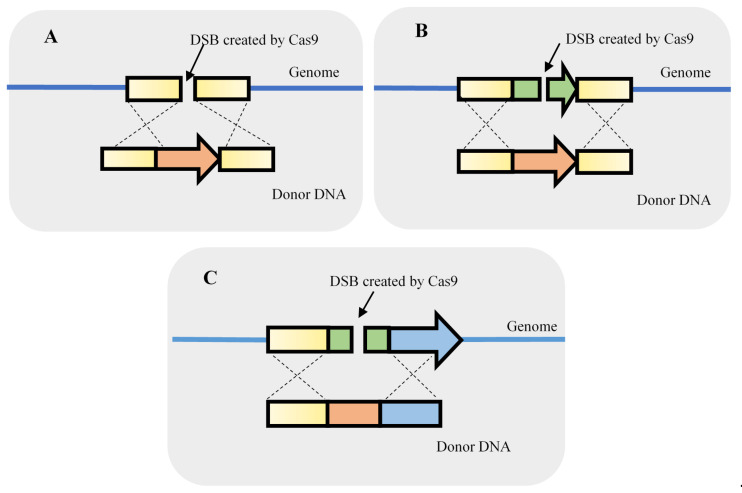
Strategies used to integrate DNA fragments using double-strand breaks (DSB) originated by clustered, regularly interspaced, short palindromic repeats–associated Cas9 (CRISPR-Cas9). The knockin can be performed by providing a homologous repair fragment for gene integration (**A**), a gene replacement (**B**), or the replacement of regulatory elements such as a promoter (**C**).

As mentioned, the CRISPR-Cas9 system may be tremendously useful to knockout single or even multiple genes without the requirement of a selection marker. The knockin of genes or other genomic components is also an important element in *S. cerevisiae* genetic studies. The knockin of genetic parts is useful both for the development of yeast cell factories and for fundamental studies. Therefore, knockin events may include the integration of heterologous genes, the replacement of genes by a feedback-insensitive orthologue or the promoter replacement to adjust the expression of a specific gene (Figure 4).

**Figure 5:**
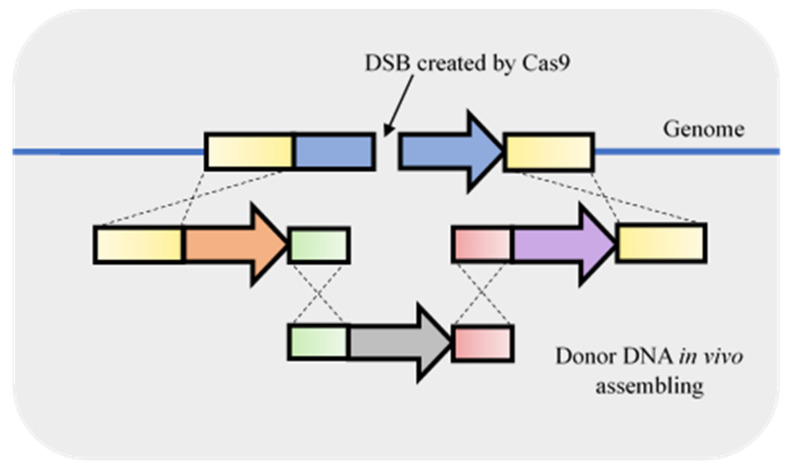
Combination of in vivo recombination and fragment integration using double-strand breaks (DSB) originated by clustered, regularly short palindromic repeats–associated Cas9 (CRISPR-Cas9). The homologous repair fragment can be in vivo assembled using *S. cerevisiae* machinery by providing the fragments with homologous ends. The assembled fragment is used to homology repair the DSB.

Combining gene disruption with the integration of a desired component may be advantageous since it makes it possible to perform various modifications in a single step. For example, Mans et al. [16] developed a CRISPR-Cas9 system to target an undesired gene and simultaneously transform the donor DNA repair with other five DNA fragments with homologous arms to in vivo combine the fragments and replace the undesired ORF. This kind of approach (Figure 5) is very useful for the development of yeast cell factories as discussed below.

### 3.2. Cell Factory Development 

The design and construction of microbial cell factories is more than ever an attractive approach to develop bioengineering processes to produce chemicals, fuels, or pharmaceutics. Furthermore, *S. cerevisiae* is an appealing model for bioengineering processes because allied with its rapid production cycles, it is easy to maintain, to manipulate, and is suitable for large-scale fermentation. The molecular “tools” available have helped the researchers to make yeast cell factories increasingly adapted to produce a desired product [46]. The development of efficient cell factories is associated with several genetic modifications, since not only the insertion of the heterologous genes is required but also the rewiring of the carbon flux toward the precursor synthesis or even other convenient modifications to improve the process. The CRISPR-Cas9 system appeared as a “Swiss army knife” [16] in the researcher’s molecular toolbox because it allowed the performance of several genetic modifications in a fast and efficient way. Furthermore, the requirement of a constant selective pressure during fermentation is negated since CRISPR-Cas9 allows a markerless genetic manipulation. In addition, several design–build–test cycles are always necessary, which increases the number of modifications required [6,7]. By using CRISPR-Cas9 systems, genetic editing has become multiplex possible, which may significantly decrease the time to perform the designed genetic modifications for a cell factory construction. The flexibility of CRISPR-Cas9 allied with the researcher’s inventiveness resulted in the development of systems that facilitate and accelerate metabolic engineering processes. Researchers are not only focused on developing new CRISPR-based strategies for direct genome engineering but also on applying CRISPR-Cas9 to improve other conventional molecular biology lab practices, speeding up the strain building process. For example, Li et al. [47] developed a CRISPR system capable of sequential rounds of gene targeting with simultaneous gRNA plasmid curing mediated by CRISPR-Cas9. Moreover, in Zhang et al.’s work [44], the developed CRISPR method skips the *Escherichia coli* transformation and verification steps. The flexibility and simplicity of the CRISPR-Cas9 system made it well accepted by genetic engineers for the development of yeast cell factories (Table 1).

**Figure 6:**
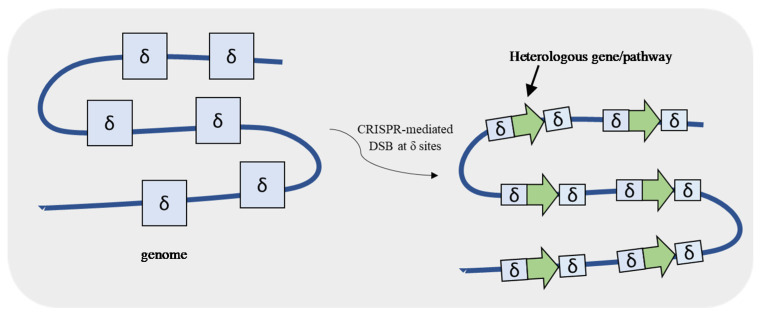
Clustered, regularly interspaced, short palindromic repeats–associated Cas9 (CRISPR-Cas9) associated with delta (δ) integration. CRISPR-Cas9 system creates a double-strand break (DSB) at the repetitive δ sequences present in the *S. cerevisiae* genome allowing the multi-copy integration of a DNA fragment containing a gene or even a pathway.

For genetic engineering purposes, the use of plasmids brings the advantage of expressing a heterologous protein/pathway in a multi-copy way once a 2µ plasmid is maintained at 10–50 copies per cell. However, genome integration has several benefits over plasmids because it allows gene expression stability and lower heterogeneity and eliminates the requirement of using selective growth media after confirmation. Shi et al. [48] developed a CRISPR-based method that allows a multi-integration of a heterologous fragment at the Ty retrotransposon, a set of repetitive sequences (delta (δ) sequences) in the yeast genome (Figure 6). The introduction of Cas9-mediated DSBs at δ sites allowed the integration of eighteen copies of a 24 kb DNA fragment carrying a xylose utilization pathway and a butanediol production pathway. The high copy number of the pathways resulted in both higher xylose consumption and butanediol production by the engineered strain comparatively to the one copy integration one.

Nevertheless, some limitations of CRISPR-Cas9 are recognized namely the efficiency variations between different targeted sites [60] and the already mentioned problems around the polyploid yeast strains. Moreover, the number of multiplex modifications is still limited and some further optimizations are recommended by Zhang et al. [44], namely the identification of stronger RNA polymerase III promoters, the optimization of plasmid construction tools to accept multiple fragments with repetitive sequences, and the development of new software that correlates the gRNA sequences with gRNA efficiencies.

### 3.3. Innovative CRISPR Toolkits in Saccharomyces cerevisiae

New emergent procedures into how CRISPR-Cas9 is applied have recently been developed demonstrating the potentialities of this tool. The target specificity of CRISPR-Cas9 systems allowed researchers to develop technologies that take advantage of its precise DNA targeting namely for the delivery of other molecules. EvolvR, for instance, combines the target specificity of CRISPR-Cas9 technology with the error-prone capacity of a mutant DNA polymerase for in vivo targeted nucleotide diversification [61]. The system employs the use of a nicking variant of Cas9 (nCas9) that cuts only one DNA strand avoiding native homology repair, and the DNA polymerase uses the nick as a start point to initiate mutation insertion. This technology was recently applied in *S. cerevisiae,* yEvolvR [62]. The results demonstrated that yEvolvR was able to insert random mutations in both directions of the target sequence, and in addition, it was possible to target two genomic loci at the same time. This technology could be important for fundamental eukaryotic research such as protein function or protein interactions or to investigate genetic mechanisms, where yeast is usually used as a role model. Moreover, it could be applied to strain tolerance engineering for industrial purposes. Engineering yeasts to confer them with increased tolerance to an external stress, such as temperature or oxidation, is a very valuable strategy to improve industrial strains’ performance. CRISPR has already been implemented for random mutagenesis via genome shuffling. Mitsui et al. [63] applied CRISPR-Cas9 for cleaving the δ-sequences in order to fragment the chromosome. During the repair of DNA fragments, large-scale modifications, such as gene amplification, translocation, and deletion, may occur. In this case, the DNA repair was induced under thermal stress conditions. After DNA repair, the modified yeast was able to grow at 39 °C and reported higher ethanol and acid resistance than the parental strain.

**Figure 7:**
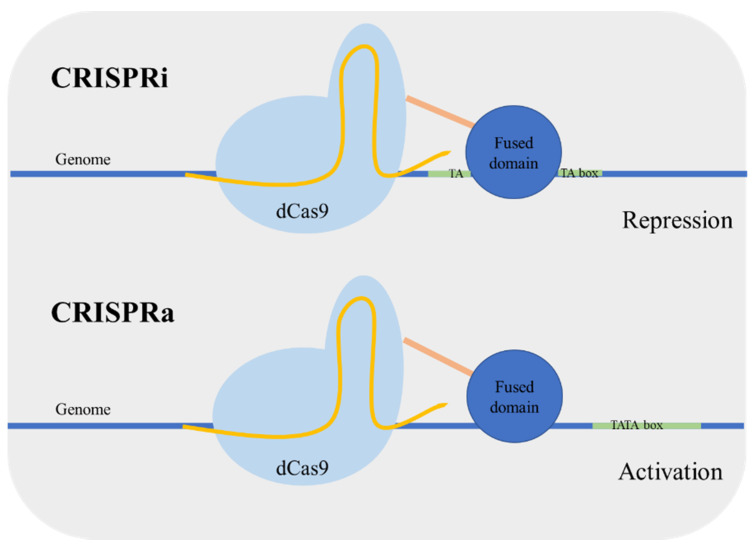
Clustered, regularly interspaced, short palindromic repeats—interference (CRISPRi) and CRISPR—activation (CRISPRa) mechanisms. CRISPRi is used for gene repression: the dead Cas9 (dCas9) with a fused domain targets the proximity of TATA box interfering with the formation of the transcriptional initiation complex and consequently repressing the gene expression. CRISPRa is used for gene activation: dCas9 fused to an activator domain targets outside the vicinity of the TATA box resulting in increased gene expression.

Targeted regulation of gene expression is important both in the context of metabolic engineering and functional genomics [34]. In yeast, the control of genetic expression is usually performed using characterized gene promoters with different strengths; however the prediction of the expression level remains a challenge. Qi et al. [64] developed an enzymatic version of Cas9 mutated in the nuclease nucleotide sites designated as dead Cas9 (dCas9). This Cas9 mutant is defective in DNA cleavage, and it can act as a simple specific DNA binding complex. Using this version of Cas9 for targeting a coding sequence caused transcriptional gene repression in *Escherichia coli*. The dCas9 binds to the target sequence blocking the action of RNA polymerase. This approach was named CRISPR interference (CRISPRi). Next, the same research group applied the system for gene repression in *S. cerevisiae* [65]. The dCas9 was guided to a specific promoter, resulting in an efficient gene repression. In addition, the repression can be enhanced by fusing a transcriptional repressor domain to dCas9. Alternatively, Farzadfard et al. [66] fused dCas9 to an activator domain and reported an activation or repression depending on the targeting site. When the target was outside the TATA box, the promoter was activated (CRISPR activation (CRISPRa)), targeting adjacent to the TATA box resulted in gene repression (CRISPRi) (Figure 7). Other activator-domain-fused dCas9 proteins were also developed achieving higher regulation levels [67]. Moreover, the CRISPRa/i system has been applied in a polyploid yeast strain, which can be very valuable for the development of more robust industrial stains [68]. 

**Figure 8:**
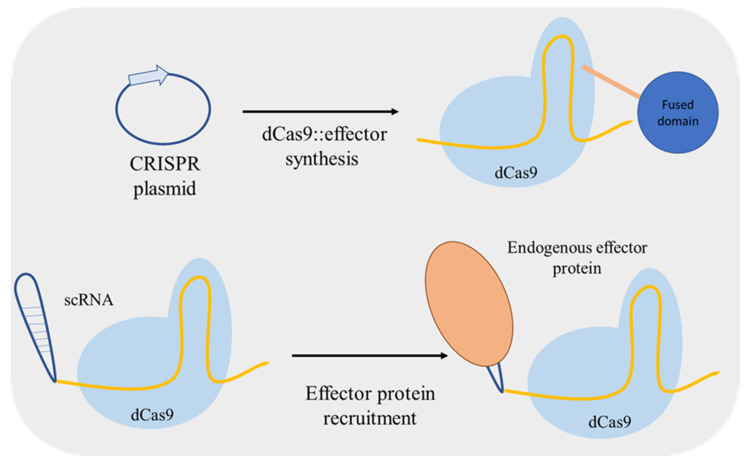
Mechanism used to associate an effector protein with dead Cas9 (dCas9). The effector molecule sequences can be fused to the dCas9 sequence and the fusion protein is expressed in *S. cerevisiae.* Otherwise, endogenous *S. cerevisiae* effector molecules can be recruited by extending guide RNAs to include RNA-based effector protein recruitment sites (scaffold RNA—scRNA), which is advantageous for repressing and activating multiple genes simultaneously. CRISPR: clustered, regularly interspaced, short palindromic repeats.

Zalatan et al. [69] used a different approach for up/downregulation of a target gene. Instead of including fusion domains in dCas9, the authors included effector protein recruitment RNA domains into the gRNA converting it to a scaffold RNA (scRNA) (Figure 8). The RNA hairpins of scRNA can recruit a specific RNA-binding protein, an activator or a repressor, thus used for locus-specific regulation. Furthermore, Jensen et al. [70] compared the regulatory performance of two distinct dCas9-mediated systems: using an inducible gRNA expression and dCas9 fused with a repressor or an activator domain; and with constitutive expression of scRNAs for effector molecule recruiting. The two systems mediated similar changes in activation/repression of the targeted promoters both at single and at multiplex level.

Moreover, a grade modulation of genetic expression was reported by Deaner and Alper [58]. The range of genetic expression was related to the proximity of dCas9-based regulators to the core of the promoter. The grade modulation applicability represents a step forward to enable a fine tuning of metabolic pathways in *S. cerevisiae*. The possible applications of CRISPRi/a are massive, and they have already been used to improve a yeast cell factory to produce β-amyrin [71].

Another possible approach, besides the use of a modified CRISPR protein, to guide effector molecules without creating a DSB, is by adjusting the gRNA molecule length. Truncated gRNAs, usually of 14 nt, proved to be able to guide the binding of Cas9 to the target sequence without the introduction of a DSB [72,73]. Hereupon, truncated gRNAs can be used for transcriptional regulation and, simultaneously, full-length gRNAs can be used for genome editing using a single Cas9 protein. This feature allowed researchers to develop multifunctional systems capable of one-pot CRISPRi, CRISPRa, or CRISPR editing. Lian et al. [74] reported the first trifunctional CRISPR system for simultaneous gene inactivation, activation, and editing in *S. cerevisiae* using truncated gRNAs for CRISPRa and CRISPRi, called CRISPR-AID. However, to avoid competition between gRNAs for the same Cas9 protein, three different PAM-recognizing CRISPR proteins were used. More recently, Dong et al. [59] established a trifunctional CRISPR system using a single Cas9 protein. The system was named CRISPR-ARE and employed the use of a Cas9 fused to a VP64-p65-Rta (VPR) activation domain. The authors demonstrated the applicability of CRISPR-ARE by optimizing α-santalene biosynthesis. The system used truncated gRNAs to target the genes for activation and repression and full-length gRNAs to edit one gene by transforming together the donor repair DNA. The editing efficiency was 100% and, gene activation and gene repression were confirmed using a reporter protein. Regarding the α-santalene biosynthesis, it increased 2.66-fold. 

## 4. Conclusions

In summary, researchers have been taking advantage of the specificity of the CRISPR-Cas9 system, not only to perform direct genetic modifications but also to direct other molecules (activators or DNA polymerases) to a previously defined locus. Engineering Cas9 protein (dCas9 or nCas9) allowed the targeting of specific sequences without introducing a DSB, which can be used to guide these molecules. Moreover, the use of truncated gRNAs allowed the development of multifunctional CRISPR systems, such CRISPR-AID and CRISPR-ARE, which will facilitate fast and multifunctional engineering of the *S. cerevisiae* chassis. 

Key Messages: The CRISPR-Cas9 system’s discovery is with no doubt a landmark in the synthetic biology field. The simplicity and flexibility of the system enabled the researchers to develop a panoply of CRISPR applications. Consequently, it is now possible to build more and more complex designs in yeast by multiplexed CRISPR-Cas9 genome editing. Besides allowing multiple modifications, the emergence of CRISPR-Cas9 enabled the researchers to perform genetic modifications faster, cheaper, and more efficiently. The possibility to perform genetic modifications without the requirement of selective markers or the multiplex genomic edits are examples of the possibilities brought by this technology. The scientific community is taking advantage of the fantastic teamwork between CRISPR and yeast with results ranging from the introduction of large chromosomal segments to point mutations and by developing improved genome editing systems. Moreover, CRISPR applications have transgressed the conventional genome editing application through the creation of novel mutated versions of Cas9 such as nCas9 or dCas9. CRISPRi/a is a tremendously useful tool for transcriptional regulation because it allows to balance and optimize gene expression without genome editing. This tool may be tremendously useful when dealing with essential genes in *S. cerevisiae* mainly for the development of industrial strains where the metabolic fluxes need to be meticulously balanced. The application of multifunctional systems, such as CRISPR-ARE, accelerates the design–build–test processes and consequently the productivity of the research. The EvolvR technology may facilitate the development of highly adapted *S. cerevisiae* strains or even simplify the study of the eukaryotic genetic mechanisms. Finally, we believe that various applications of the CRISPR-Cas9 systems in *S. cerevisiae* will continue to evolve in order to respond to the challenges of the biotechnological field and to contribute to the sustainability of the bio-based industry.

## Figures and Tables

**Table 1:** Some CRISPR-based applications in *Saccharomyces cerevisiae*. ARS: autonomously replicating sequence; CEN: yeast centromere.

Objective	Cas Protein Expression System	gRNA Expression	Phenotype Desired	Transformed Elements	Number of Editions	Ref.
Genome editing	CEN plasmid	pSNR52	Canavanine resistance	CRISPR-Cas9 plasmid	1	[14]
Genome editing	2µ plasmid	pSNR52 plus CRISPR native array	Hydrocortisone production	CRISPR-Cas9 plasmid + Donor DNA	3	[15]
Genome editing	2µ plasmid	pSNR52 with 5’ HDV	Cellobiose utilization	CRISPR-Cas9 plasmid + Donor DNA	3	[19]
Genome editing	Integrated	pSNR52	Muconic acid production	gRNA plasmid + Donor DNA	3	[49]
Genome editing	2µ plasmid	pSNR52 tRNA–gRNA array	Increase free fatty acid production	CRISPR-Cas9 plasmid	8	[44]
Genome editing	CEN plasmid	pSNR52	Mevalonate production	CRISPR-Cas9 plasmid + Donor oligos	5	[21]
Genome editing	2µ plasmid	pSNR52	Leucine and Isoleucine auxotrophy	CRISPR-Cas9 plasmid + Donor oligos	2	[17]
Genome insertion	2µ plasmid	pSNR52	BDO production; Xylose utilization	CRISPR-Cas9 plasmid + Donor DNA	Pathway insertion	[48]
Genome insertion	2µ plasmid	ptRNA	Taxadiene production	CRISPR-Cas9 plasmid + Donor DNA	4	[23]
Genome insertion	CEN plasmid	pSNR52	β-carotene production	Cas9 plasmid + gRNA plasmid + Donor DNA	3	[25]
Genome editing and insertion	Cas9 integration	pSNR52	*p*-coumaric acid production	gRNA plasmid	10	[50]
Genome editing	2µ plasmid	pSNR52 plus CRISPR native array	Increase ethanol production	CRISPR-Cas9 plasmid + Donor DNA plasmid	3	[43]
Genome editing and insertion	2µ plasmid	pSNR52	Enhance synthesis of farnesyl diphosphate	Cas9 plasmid + gRNA plasmids + Donor DNA	5	[51]
Genome editing and insertion	2µ plasmid	pSNR52	Enhance fatty acid production	Cas9 plasmid + gRNA plasmids + Donor DNA	4	[52]
Genome editing and insertion	2µ plasmid	pSNR52	Butanediol production	Cas9 plasmid + gRNA plasmids + Donor DNA	5	[53]
Genome insertion	2µ plasmid	pSNR52	Minimize ethyl carbamate accumulation	CRISPR-Cas9 plasmid +	1	[54]
Genome editing and integration	Integrated	pSNR52	Production of monoterpene precursor, geraniol	gRNA plasmid + Donor DNA	8	[55]
Genome editing and integration	CEN plasmid	pSNR52	Limonene production	Cas9 plasmid + gRNA plasmid + Donor DNA	9	[56]
Genome editing and integration	Integrated	pSNR52	2-phenylethanol production	gRNA plasmid + Donor DNA	8	[57]
Gene activation and repression	ARS/CEN plasmid (dCas9 fused with VPR domain)	pSNR52	Optimize isoprenoids and triacylglycerols biosynthesis	dCas9 plasmid + gRNA plasmid	4	[58]
Gene activation and repression	ARS/CEN plasmid (Cas9 fused with VPR domain)	pSNR52	Optimize α-santalene biosynthesis	Cas9 plasmid + truncated gRNA plasmid	3	[59]
